# A Narrative Review of Nonpharmacological Approaches to Control Anxiety in Pediatric Dentistry

**DOI:** 10.1155/ijpe/6139102

**Published:** 2025-09-27

**Authors:** Sepehr Siahvoshi, Sahar Hassantash, Katayoun Salem

**Affiliations:** ^1^Dental Materials Research Center, Dental School, Islamic Azad University of Medical Sciences, Tehran, Iran; ^2^Public Health Department, University of Miami, Miami, Florida, USA; ^3^Department of Pediatric Dentistry, TeMS.C., Islamic Azad University, Tehran, Iran

**Keywords:** child, dental anxiety, dental fear, pediatric dentistry

## Abstract

**Objectives:** This review critically evaluates the literature on nonpharmacological interventions aimed at reducing anxiety in pediatric patients undergoing dental treatments. The aim is to analyze recent research findings on various behavior management techniques and their clinical implications.

**Methods:** A thorough literature search was conducted using electronic databases, including PubMed, Web of Science, the Cochrane Library, and Scopus. Predefined terms were used to ensure a comprehensive and focused search for relevant studies.

**Results:** The continuous stream of research has unveiled several methods, such as awards, Tell–Show–Do, exposure to positive images, and pictorial stories, that have demonstrated tangible effects on anxiety, behavior, and pain perception. These interventions have exhibited practical effectiveness in actual clinical settings.

**Conclusions:** The findings underscore the importance of implementing nonpharmacological interventions in pediatric dentistry to establish a dependable, reassuring, and efficacious treatment environment for children. As more research adhering to designated guidelines and methodologies emerges, pediatric dentistry can further enhance its capacity to not only reduce dental fear and pain intensity but also promote positive dental hygiene practices, starting from early childhood.

## 1. Introduction

Dental anxiety and fear commonly manifest during childhood and are considered characteristics of the developmental process. However, the subsequent avoidance of receiving dental treatment may result in a detrimental cycle and can exacerbate oral health issues and a lower quality of life [1–3]. Dental anxiety is associated with infrequent dental visits, reduced child cooperation, and higher rates of dental caries, making it a critical concern in oral healthcare [4, 5]. In severe cases, children with dental phobia may refuse treatment even when experiencing pain that could be alleviated by appropriate care [6].

Estimates of dental anxiety prevalence among children and adolescents range from 5.7% to 42.4% [2, 7–11], and its occurrence seems influenced by various factors, including sex, age, cultural context, maternal characteristics, socioeconomic status, presence of dental caries, history of toothaches, previous dental treatments, and parenting attitudes [12–17]. Epidemiological studies provide data on the prevalence of dental anxiety. For instance, in the Netherlands, the predicted burden of disability-adjusted life years (DALYs) attributed to dental anxiety amounts to 73,000, ranking it 16th nationwide [18]. Systematic reviews and meta-analyses provide robust evidence on the global prevalence of dental fear and anxiety (DFA) in pediatric populations. Grisolia et al. [2] conducted a comprehensive review of 49 observational studies, reporting global age-specific prevalence rates of dental anxiety as follows: 35.5% in preschoolers, 24.8% in schoolchildren, and 13.3% in adolescents.

Additionally, a more recent systematic review designed by Sun et al. [19] focusing on early childhood (ages 2–6) estimated a pooled prevalence of DFA at 30% (95% CI: 25–35), with higher odds of anxiety among children without prior dental visit experience (OR = 1.36, 95% CI: 1.18–1.58) and those with caries experience (OR = 1.18, 95% CI: 1.09–1.26). These findings indicate that dental anxiety is a pervasive issue, particularly among younger children, and is influenced by factors such as lack of familiarity with dental settings and existing oral health conditions.

Positive experiences have demonstrated efficacy in reducing dental anxiety and improving children's quality of life in terms of oral health [20]. Children often struggle to manage their behavior due to limited communication skills and an inability to deal with threatening dental stimuli. Identification and stress management are therefore key to effective treatment [21].

“Although the dental procedure may be perfect, the appointment is not a success if the child leaves in tears,” McElroy stated in 1895 [22]. While some recommend using mild and moderate, or deep sedation to manage anxiety [23], pharmacological interventions require costly resources, pose risks and adverse effects, and demand careful management, monitoring, and documentation [24, 25]. Various psychological treatments have emerged as imperative in pediatric dentistry settings, particularly as parental consent for conscious sedation or general anesthesia becomes less favorable. The healthcare practitioner will ascertain the most effective behavioral management technique for each patient based on an initial assessment of their anxiety level [26].

Recent studies have explored the efficacy of behavior management techniques in dental treatment incorporating distraction, visualization, persuasion, and innovative approaches in addition to traditional methods [27]. Techniques such as positive image viewing, virtual reality (VR), music therapy, and magic tricks demonstrate promise in improving compliance, child behavior, and reducing anxiety and pain perception [28–30]. Such investigations must be carried out using appropriate methodologies, aiming to provide reliable answers about their efficacy and supporting their application in pediatric dentistry. This article is aimed at evaluating the existing literature on the effects of nonpharmacologic interventions for anxiety, behavior, and pain perception in pediatric dentistry.

## 2. Materials and Methods

A comprehensive electronic literature search was conducted across PubMed, Web of Science, the Cochrane Library, and Scopus for articles published from 1885 to 2025 using a set of predefined terms. The search strategies encompassed the following keywords: (Dental Anxiety OR Dental Anxieties OR Anxiety, Dental OR Dental Phobia OR Fear, Dental OR Dentophobia OR Dentophobias OR Odontophobia OR Odontophobias OR Dental Fear OR Dental Fears OR Phobia, Dental OR Dental Phobias) AND (Anti-Anxiety Effect OR Anti Anxiety Effect OR Effect, Anti-Anxiety OR Anxiolytic Effects OR Effects, Anxiolytic OR Antianxiety Effects OR Effects, Antianxiety OR Anxiolytic Effect OR Effect, Anxiolytic OR Anti-Anxiety Effects OR Anti Anxiety Effects OR Effects, Anti-Anxiety OR Antianxiety Effect OR Effect, Antianxiety) AND (Pediatric Dentistry OR Pedodontics OR Dentistry, Pediatric).

The following were the criteria for inclusion: studies that address the effectiveness of any nonpharmacological technique for anxiety reduction in pediatric dentistry. Opinion articles, commentaries, editorials, and conference abstracts were not considered.

**Figure 1:**
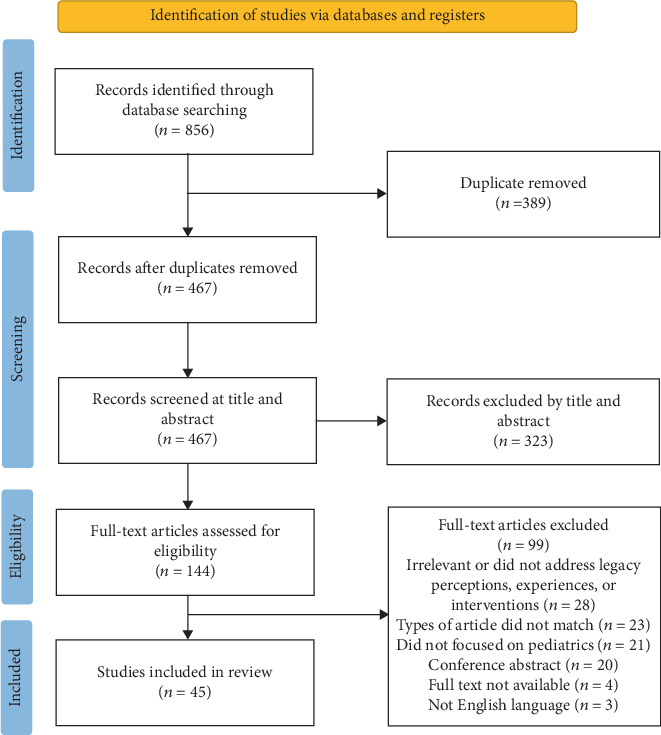
PRISMA flow chart showing the inclusion and exclusion criteria.

Following the removal of duplicate records, titles and abstracts were screened, and studies meeting the criteria underwent full-text review for inclusion in this review.  Figure 1 presents a schematic diagram of the identification and selection process for the included studies in the analysis.

### 2.1. Audiovisual Distraction

Previous research studies have shown that using a distraction technique during dental procedures benefits patients by reducing their level of distress, which in turn lowers their perception of pain, particularly during local anesthetic injections [31–34]. Widely accepted by parents, audiovisual distraction techniques demonstrated superior effectiveness compared to audio distraction alone in managing stress in pediatric dental patients [35]. Studies indicated positive outcomes using video games as a form of positive distraction, proving more effective in reducing dental anxiety compared to video cartoons among school children [36, 37]. Additionally, employing audiovisual storytelling exhibited stress reduction benefits, recommended for use by pediatric dentists [38].

### 2.2. Virtual Reality 

In VR, users are tricked into the illusion of being immersed inside a three-dimensional (3D) setting using technology that allows them to interact with computers in a more natural way [39]. Rao et al. [40] and Raseena et al. [41] involved children utilizing VR eyeglasses, revealing that audiovisual distraction via VR significantly reduced the FACES Pain Scale and anxiety levels. Furthermore, Vabitha et al. [42] observed a substantial decrease in salivary cortisol levels among children who wore VR goggles showing improved behavior during procedures like caries removal and restorative treatments [43]. Accordingly, Pathak et al. [44] evidenced by heart rate (HR) measurements that using VR technology in children reduced anxiety during the extraction of primary molars in children. VR glasses, along with distraction techniques, were found to be more effective at reducing dental anxiety among children aged 5–8 years during dental treatments than preoperative use of dental simulation games [45]. Robertson et al. [46] affirmed the absence of contraindications for employing distraction techniques in pediatric dentistry, emphasizing the positive impact of such methods in alleviating anxiety. A study conducted in Iran, using a split-mouth randomized crossover clinical trial design, investigated the effects of VR headsets on pain and anxiety levels in 6–8-year-old children undergoing primary mandibular pulpotomy. Pulse rate (PR) and the Modified Child Dental Anxiety Scale (MCDAS) were used as outcome measures. The study results suggest that VR headsets can be effective in reducing both pain and anxiety during this dental procedure [47].

### 2.3. Music Distraction

In 1959, Gardner and Licklider introduced the concept of audio analgesia, employing audio techniques to reduce pain without pharmacological agents commonly applied during painful medical treatments, such as dental treatment procedures [48]. Techniques involving live music, well-known songs, white noise, and relaxing sounds have historically served as effective distractions, demonstrating a capacity to diminish DA and subsequently elevate pain thresholds among patients [49–51]. However, controversy surrounds the effectiveness of this method. According to Gupta et al. [52] findings, anxiety, pain, or disruptive behavior were not decreased by the audio music distraction approach. Similarly, Aitken et al. [50] observed no significant differences in anxiety or pain perception when comparing upbeat and relaxing music distractions to a control group (no music) during restorative treatments. On the contrary, in another study, instrumental music could reduce levels of anxiety in pediatric patients during various treatment sessions, albeit not remarkably significant. Assessing anxiety using tools like the Venham picture test (VPT), Venham's clinical anxiety rating scale (VCRS), oxygen saturation, and PR [29], Jindal and Kaur [53] noted that during the third and fourth visits for restorative and invasive procedures, anxious patients experienced a significant reduction in anxiety due to audio distraction with VPT. Singh et al. [54] research further corroborated the positive impact of music on controlling the anxiety of pediatric patients, evidenced by measures such as PR, VPT scores, and systolic blood pressure. Alkahtani et al. [55] similarly emphasized the positive effects of music in reducing DA. Using binaural beats, which combine the advantages of traditional audio distraction with brainwave entrainment, effectively decreases pre- and intraoperative DA in pediatric patients by presenting two steady-intensity sounds with differing frequencies separately to each ear [56, 57].

### 2.4. Magic Tricks

Thaumaturgy, or magic tricks, has been shown to reduce anxiety by altering the perception of unpleasant experiences. This approach has proven valuable in enhancing self-confidence among patients with psychological issues, notably in pediatric oncology where it has been employed to reduce anxiety during injections [58]. Studies have demonstrated the effectiveness of magic tricks in pediatric settings, not only to relax children but also to foster a positive relationship between the child and the dentist. The interactive nature of thaumaturgy, as seen in tricks like the “thumb and light” trick, engages the child's curiosity and attention, providing a distraction that allows dental procedures to be performed more easily [58].

Peretz and Gluck [30] pioneered the use of magic tricks as a behavioral management technique in pediatric dentistry, particularly for taking radiographs. They highlighted the importance of cognitive development in the effectiveness of these techniques. According to Konde et al. [59], thaumaturgy describes a variety of tricks that can be used to shape the behavior of uncooperative children, regarding their age and cognitive development. In the study by Thosar et al. [60], the effectiveness of using magic tricks and audiovisual aids in reducing children's anxiety was examined and compared. The results revealed that both techniques had comparable positive effects on decreasing children's stress during dental treatment.

Kothari et al. in a randomized controlled trial in India demonstrated that the use of magic tricks significantly alleviated anxiety related to inferior alveolar nerve blocks (IANBs) in young children. This cost-effective and risk-free intervention yielded measurable benefits, although the extent of these benefits may vary among individual children [61].

### 2.5. Modeling

In 1967, Bandura defined modeling as acquiring behavior through observation, which can reduce children's fear and avoidance behavior by encouraging them to replicate the observed actions in similar situations [62, 63]. Modeling can be performed live, using peers, siblings, or parents, or through filmed methods. The first studies on modeling in pediatric dentistry were conducted in the late 1960s, and research since then has demonstrated its therapeutic effect in reducing anxiety and improving coping skills in children during medical situations [64–66]. Farhat-McHayleh et al. [67] suggested using live modeling and stated that the model used (e.g., mother or father) and the age of the child are determining factors in the success of this technique. Nevertheless, both live and filmed modeling have proven effective, with filmed modeling showing similar success to desensitization techniques. It has the added advantage of being time-efficient for the dental team. A randomized clinical trial by Paryab and Arab [63] have confirmed that filmed modeling is as effective as the commonly used Tell–Show–Do (TSD) technique in reducing anxiety and increasing cooperative behavior in children aged 4–6 years during dental treatment, with no significant differences in anxiety, HR, or cooperation scores. Another study by Sahebalam et al. [68] demonstrated the effectiveness of an animated-modeling approach, which significantly lowered anxiety and increased cooperation levels compared to the TSD method during two dental visits for children aged 4–6. Additionally, a comparative study by Kevadia et al. [69] evaluated three behavior modification techniques—Tell-Play-Do (TPD), filmed modeling, and a smartphone dental app—among children aged 6–9 years during dental procedures. The study found that while all methods were effective, the TPD technique resulted in the lowest HR, facial image scale (FIS), and Venham's pictorial index (VPI) scores, making it more effective in reducing children's anxiety and fear during dental treatment compared to filmed modeling and the smartphone app. This highlights that filmed or animated modeling, while beneficial, can be further enhanced or complemented by alternative techniques like TPD.

### 2.6. TSD

TSD initially introduced by Addlestone in 1959, closely resembles desensitization, involving a gradual exposure of young patients to a dental procedure, allowing them to assimilate the process gradually [70]. This method entails an initial explanation of the procedure in terms that the child can comprehend, followed by a demonstration that engages relevant senses. Upon the child's acceptance of the approach, reinforcing positive behavior becomes integral, thereby incorporating the technique into behavior shaping [71]. This approach is on the basis of learning theory and combines positive reinforcement with both verbal and nonverbal communication skills [72, 73].

To expand on this approach, children can engage with dental imitation instrument toys that offer a more comprehensive understanding rather than solely observing a model or instructions. This concept led to the TPD technique, which reduced anxiety and fear in children by utilizing the principle of learning. Lavanya et al. and Vishwakarma et al. showed that the TPD technique reduced HR in 5–7-year-old children, increased cooperation during dental treatment, and was more effective than live modeling at reducing child anxiety levels [63, 74, 75].

### 2.7. Animal-Assisted Therapy (AAT)

As an adjunct to other strategies, AAT is an applied interdisciplinary technique that uses animals to help patients with resolving their behavioral problems [76]. According to the studies of Gupta and Yadav [77], this interdisciplinary technique involves the interaction between a specially trained animal for this approach and the patient, which has improved the behavior among eligible patients. In 1969, Dr. Boris M. Levinson notably introduced his dog, Jingles, as the first documented animal therapy dog, intending to assist withdrawn children [78]. According to the statements of Charowski et al. [79], pediatric users of this therapy reported high satisfaction levels, but anxiety levels in both control and experimental groups were the same. Conversely, the Nalini et al. [80] studies revealed that dogs were found to be the most popular pet for very young children; 57.5% of children preferred live pets, and AAT was found to be helpful in reducing dental anxiety in children. In a study by Gussgard et al. [81] on 6–12-year-old pediatric patients with anticipatory anxiety and situational fear, the presence of a dental therapy dog in an operatory room facilitated intraoral clinical examinations.

### 2.8. Hypnosis

The application of hypnosis has demonstrated a reduction in postoperative pain linked to conscious sedation [82]. This method entailed the use of ocular fixation and verbal cues. Throughout the process of hypnotic induction, the hypnotist meticulously directed patients through serene, soothing, or pleasant visualizations to enhance their comfort, divert their attention from adverse stimuli, and increase their susceptibility to therapeutic suggestions. The deepening phase incorporated imagery of natural scenes or narrative techniques, and analgesia was achieved through specific conditioning administered 23 h prior to the patient's emergence from hypnosis. In children receiving local anesthesia for pulp therapy, it has been proven effective in lowering HR and physical resistance and reducing children's discomfort during tooth extractions [83, 84].

Furthermore, hypnosis assists children in better pain management even following more distressing procedures like tooth extraction, potentially minimizing the need for analgesic medications [85]. Upon comparing hypnosis with pharmacological methods of anxiety reduction, including nitrous oxide, hypnosis was evaluated as beneficial. According to the study by Motallebi et al. [86], and based on the fact that the incidence of pain was reported to be lower in the hypnosis group compared to the group under sedation with nitrous oxide, the superiority of hypnosis can be asserted. The analysis conclusively demonstrated that hypnosis is a highly effective tool for managing a child's behavior during primary molar pulpotomies. It significantly enhances cooperation and markedly reduces resistance [87].

### 2.9. Laughter Therapy

A positive emotional state, which can be elicited through the viewing of humorous videos or interaction with clowns prior to dental and medical procedures, has been shown to enhance pain tolerance thresholds, bolster immune responses, and mitigate adverse cardiovascular effects associated with negative emotional states [88, 89]. This natural response induces muscle relaxation, deeper and faster breathing, increased endorphin production, and a decreased secretion of stress-related hormones like corticosteroids [90]. Laughter therapy significantly reduces DA and alleviates pain and discomfort during dental procedures, according to Felluga et al. [91] and Weisenberg et al. [92]. Additionally, Vagnoli et al. [93] stated the notably positive impact of children interacting with clowns in reducing anxiety before surgery, a finding supported by Demir [90], Alcântara et al. [94], Cosi et al. [95], and Morishima et al. [96]. Jahanimoghadam et al. [97] reported that the reduction in DA due to preoperative laughter intervention was more pronounced in boys.

### 2.10. Other Techniques

Using a rubber dam, as indicated by Vanhée et al. [98], allows dentists to deliver optimal dental care while reducing DA in young patients, though it may prolong procedures and necessitate training for precise placement.

In children with both low and high DA, some studies recommend using the computerized controlled local anesthetic device (CCLAD) to administer local anesthesia rather than the conventional cartridge delivery method [99]. Research by Kotian et al. [100] suggests that incorporating kid-friendly colors like blue and yellow in dental offices may help children develop a positive attitude toward dental procedures.

Aromatherapy was proposed as another method to reduce DA, with some studies suggesting its effectiveness comparable to music intervention [101]. Abdalhai et al. [102] concluded that the combination of aromatherapy using Lavender–Neroli oil and music constitutes an effective and safe nonpharmacological approach for managing dental anxiety in pediatric patients.

Based on Rank et al.'s [103] study, receiving an award as a form of positive reinforcement after dental treatment demonstrated that preschoolers' anxiety decreased after two visits. During the second visit, girls in the experimental group exhibited lower levels of DA compared to boys.

Table 1 provides a comprehensive summary of the included studies on nonpharmacologic interventions for dental anxiety in children, detailing the various intervention types, their characteristics, study designs, populations, and key findings to facilitate a clear understanding of the approaches employed.

## 3. Conclusion

In conclusion, this comprehensive literature review underscores the significant role of nonpharmacological interventions in managing pain, anxiety, and behavioral issues in pediatric dentistry. Various methods, including awards, TSD, exposure to positive images, and pictorial stories, demonstrate tangible impacts on these essential aspects of pediatric dental care. Notably, the available evidence supports the practical effectiveness of these interventions, advocating for their adoption in clinical practice. Simultaneously, it highlights the need for ongoing, rigorous research using standardized methodologies to reinforce these findings and explore novel avenues. Adopting such a scientific approach enables the pediatric dentistry field to uphold its primary objective—the facilitation of a reliable, comforting, and effective treatment environment for children. This, in turn, significantly contributes to alleviating common concerns like dental fear and pain intensity while fostering positive dental hygiene practices from an early age.

## Figures and Tables

**Table 1:** Summary of included studies on nonpharmacologic interventions for dental anxiety in children.

**Study (author, year)**	**Study type**	**Sample characteristics**	**Intervention type**	**Instruments used**	**Main findings**
Al-Namankany et al., 2014 [31]	RCT	60 children, 5–10 years old	Video modeling	Venham's Picture Test, Facial Image Scale	Video modeling significantly reduced anxiety during dental injections.

Al-Halabi et al., 2018 [32]	RCT	60 children, 4–8 years old	VR eyeglasses vs. tablet distraction	Venham Behavior Rating Scale	VR glasses more effective in managing anxiety and improving behavior.

Shekhar, 2019 [34]	Quasi-experimental	40 children, 6–10 years old	Audiovisual distraction during LA procedures	MCDAS, pulse rate	Reduced anxiety and physiological arousal with distraction.

Viswanath & Naithani, 2014 [35]	Comparative study	60 children, 4–8 years old	Audio vs. audiovisual distraction	Facial Image Scale, pulse rate	AV distraction more effective than audio-only in reducing anxiety.

Alsibai et al., 2023 [36]	RCT	72 children, 4–9 years old	Active distraction with squeeze ball	Venham Behavior Rating Scale, pulse rate	Active distraction significantly improved cooperation.

Guinot et al., 2021 [37]	Randomized crossover trial	80 children, 4–7 years old	Active vs passive audiovisual distraction	Wong-Baker FACES, pulse rate	Active distraction resulted in better behavior and lower anxiety.

Gozin et al., 2022 [38]	RCT	60 Iranian children, 5–7 years old	Audiovisual storytelling	Facial Image Scale, pulse rate	Significant reduction in anxiety scores post-intervention.

Rao et al., 2019 [40]	Behavioral interventional study	60 children, 6–10 years old	Virtual reality distraction	Wong–Baker FACES, pulse rate	VR distraction reduced pain perception and anxiety.

Shetty et al., 2019 [42]	RCT	60 children, 5–8 years old	VR distraction	Wong–Baker FACES, pulse rate	VR significantly lowered pain and anxiety.

Pathak et al., 2023 [44]	Clinical evaluation	60 children, 5–10 years old	VR during LA and extractions	Behavior assessment, heart rate	Feasible and effective for reducing anxiety.

Baniebrahimi et al., 2022 [45]	RCT	50 children, 6–9 years old	VR vs. mobile games	Facial Image Scale, MCDAS	Both effective; VR slightly better.

Bahrololoomi et al., 2024 [47]	Randomized crossover trial	50 children, 6–9 years old	VR distraction	Wong–Baker FACES, pulse rate	Reduced anxiety and pain effectively.

Aitken et al., 2002 [50]	RCT	45 children, 4–6 years old	Upbeat vs. relaxing music vs. no music	Parent MCDAS, Venham's Scale, HR, NCBS, VAS	No significant differences in anxiety/pain/behavior; 90% enjoyed music.

Gandhi & Subramanian, 2021 [51]	Comparative study	45 children, 4–6 years old	White noise vs. nursery rhymes vs. movie songs (during N_₂_O sedation)	Venham Scale	No statistical difference (*p* > 0.05); white noise group showed best clinical compliance (70% mild distress).

Gupta et al., 2017 [52]	RCT	60 children, 3–7 years old	Upbeat music vs. relaxing music vs. no music	Venham Picture Test, NC Behavior Scale, VAS, HR	Music did not produce a reduction in pain, anxiety, or disruptive behavior.

Singh et al., 2014 [54]	RCT	60 children, 6–12 years old	Patient-selected music during extraction	Venham Picture Test, pulse rate, BP, O_₂_ sat	Significant anxiety reduction, pulse rate, SBP (*p* < 0.05); no change in O_₂_ saturation.

Alkahtani et al., 2020 [55]	Cross-sectional survey	50 children, 5–14 years old	Music distraction during dental procedures	Modified Child Dental Anxiety Scale (MCDASf)	Significant reduction in self-reported anxiety; 78% felt relaxed with music (vs 50% without). 80% reported calmness and preferred music for future visits.

Padawe et al., 2023 [57]	RCT	120 children, 3–10 years old, ASA I	Binaural beats vs. white noise	Venham Picture Test, Pulse oximeter	Significant anxiety reduction with binaural beats vs. control (*p* < 0.01).

Hart & Walton, 2010 [58]	Clinical study	Hospitalized pediatric patients (≥ 5 years old)	Therapeutic bedside magic	Observational reports, anecdotal feedback	Reduced anxiety, improved mood/coping; effective for difficult patients.

Konde et al., 2020 [59]	RCT	240 children, 2–13 years old	Age-specific magic tricks	Facial Anxiety Scale (FAS)	Age-specific tricks reduced anxiety (thumb-light: 2–7 y, book: 7–11 y, item: 11–13 y)

Thosar et al., 2022 [60]	Observational comparative study	30 children, 4–11 years old, Frankl behavior ratings 2–3	Magic trick vs. audiovisual distraction	Venham Picture Test, Modified VAS, pulse oximeter	Both techniques reduced anxiety significantly; audiovisual distraction showed greater intra-op physiological improvement.

Kothari et al., 2023 [61]	RCT	30 anxious children, 4–6 years old	Magic tricks vs. conventional methods	RMS-PS scale, Venham's scale, pulse oximeter	Significantly lower anxiety with magic tricks during nerve blocks (*p* < 0.05).

Bandura et al., 1967 [62]	Modeling experiment	Children with dog avoidance behavior	Observing fearless peer models	Avoidance measures	Vicarious exposure to models significantly reduced avoidance behavior; positive context provided no added benefit.

Paryab & Arab, 2014 [63]	RCT	46 children, 4–6 years old	Filmed modeling vs. Tell–Show–Do	Venham Scale, Frankl Index, heart rate	No significant differences in anxiety, cooperation, or heart rate between groups.

Allen et al., 1990 [65]	Survey	160 pediatric dentistry diplomates	Evaluation of 15 management practices	Practitioner survey	Traditional techniques (hand-over-mouth, tell–show–do, sedation, restraint) preferred over newer methods (modeling, contingent rewards). 25% of children posed management difficulties; need for safer alternatives identified.

Greenbaum & Melamed, 1988 [66]	Theoretical review	N/A (conceptual)	Pretreatment modeling (videotape)	N/A	Modeling reduces children's dental fear, prevents lifelong avoidance, decreases disruptive behavior, shortens treatment time, and integrates efficiently into waiting periods.

Sahebalam R et al., 2020 [68]	RCT	48 children, 4–6 years old	Animated modeling (Jilo) vs. Tell–Show–Do	Venham Anxiety (VCAS), Venham Cooperation (VCCS)	Jilo group had lower anxiety/cooperation scores vs. TSD (*p* < 0.05) in both visits.

Kevadia et al., 2020 [69]	RCT	75 children, 6–9 years old	Tell–Play–Do vs. film modeling vs. dental app	Heart rate, Facial Image Scale (FIS), Venham Pictorial	TPD most effective: lowest HR, FIS, VPI scores.

Vishwakarma et al. 2017 [75]	RCT	98 children, 5–7 years old	TPD vs. live modeling	Heart rate, Facial Image Scale (FIS), Venham Index	TPD significantly reduced anxiety (heart rate, FIS, Venham scores) vs. live modeling.

Massouda et al., 2025 [104]	Pilot prospective clinical trial	39 children aged 7-14 years old	Animal-assisted therapy (AAT)	Heart rate, Salivary cortisol and a-amylase, video coding for behavioral measures	It reduced the pain and improved behavioral responses in pediatric dental patients. While physiological measures like heart rate and behavioral observations showed positive trends, and postoperative pain was significantly reduced, self-reported anxiety and salivary stress markers did not show significant differences.

Charowski et al., 2021 [79]	RCT	47 children, 6–10 years old	AAT	Pulse, SpO_₂_, FACES, Frankl/Houpt scales	AAT improved behavior during procedural transitions despite no physiological anxiety reduction.

Gussgard et al., 2023 [81]	Pilot randomized clinical trial (RCT) with a cross-over design	16 children aged 6-12 years old	AAT (dog-assisted therapy)	CFSS-DS scale, happy–sad-face diagram, salivary cortisol level, heart rate variability, and skin conductance	Dog-assisted therapy positively affect children with dental anxiety or those who avoid dental care.

Huet et al., 2011 [83]	Prospective controlled study	29 children aged 5–12 years old	Hypnosis	Modified Yale Preoperative Anxiety Scale (mYPAS), Visual Analogue Scale (VAS), Modified Objective Pain Score (mOPS)	Hypnosis may be effective in reducing anxiety and pain in children receiving dental anesthesia.

Oberoi et al., 2016 [84]	RCT	200 children aged 6-16 years old	Hypnosis	Monitor pulse rate and oxygen saturation, physical and verbal resistance, Stanford hypnotic scale	Hypnosis reduced resistance and lower heart rates during local anesthesia administration, thereby improving patient cooperation

Sabherwal et al., 2021 [85]	RCT	60 children aged 8–12 years old	Hypnosis and progressive muscle relaxation	Visual Facial Anxiety Scale (VFAS), heart rate, blood pressure, oxygen saturation, Wong–Baker Faces Pain Rating Scale (WBS)	Both hypnosis and progressive muscle relaxation significantly reduced anxiety, heart rate, blood pressure, and postoperative pain.

Motallebi et al., 2024 [86]	RCT	66 children aged 6–10.5 years old	Hypnosis, nitrous oxide/oxygen inhalation	Venham Clinical Anxiety Scale (VCAS), Venham Picture Test (VPT), Children's Fear Survey Schedule (CFSS-DS), Modified Child Dental Anxiety Scale (faces version) (MCDAS(f)), Venham Clinical Cooperation Scale (VCCS), heart rate, oxygen saturation (SpO_2_)	Both hypnosis and nitrous oxide inhalation are effective methods for reducing anxiety and improving cooperation in school-aged children. While pain levels were similar across groups, the hypnosis group reported less pain overall.

Girón et al., 2024 [87]	RCT	60 children, aged 5–7 years old	Hypnosis	Face, Legs, Activity, Crying, Consolability (FLACC) scale, heart rate, skin conductance	Hypnosis is a more effective method than the traditional tell/show/do technique for reducing anxiety and pain in children.

Chen et al., 2024 [89]	Nonrandomized controlled trial	104 children aged 3–6 years old	Family participatory clown therapy	FLACC scale, Visual Analogue Scale for Anxiety (VAS-A), State Anxiety Inventory (S-AI), Children's Fear Scale (CFS), Crying incidence, venipuncture success rate, child compliance, and parental satisfaction	Family participatory clown therapy reduces pain, anxiety, medical fear, and crying and improves venipuncture compliance and increases parental satisfaction.

Felluga et al., 2016 [91]	Quasi-randomized controlled trial	40 children 4–11 years old	Clown therapy	Children's Anxiety and Pain Scales (CAPS-Anxiety), Numerical Rating Scale (NRS), Clown Effectiveness Self-Evaluation Form	Clown therapy reduces children's anxiety during painful procedures but does not significantly impact pain levels.

Vagnoli et al., 2005 [93]	Randomized, prospective study	40 children 5–12 years old	Clown therapy	Modified Yale Preoperative Anxiety Scale (m-YPAS), State-Trait Anxiety Inventory (STAI), and Clown Self-Evaluation	The presence of clown doctors, alongside a parent, is an effective intervention for managing children's preoperative anxiety during anesthesia induction.

Jahanimoghadam et al., 2022 [97]	Double-blinded RCT	48 children aged 5–7 years old	Laughter therapy	Modified Child Dental Anxiety Scale Faces questionnaire (MCDASF), Wong–Baker Faces Scale, Sound, Eye, Motor (SEM) scale, Screen for Child Anxiety Related Disorder questionnaire (SCARED)	Laughter intervention prior to dental procedures significantly reduces both anxiety and pain in pediatric patients, with a more pronounced effect observed in boys. This suggests laughter therapy is a valuable, low-cost intervention for managing dental distress in children.

Vanhee et al., 2021 [98]	RCT	51 children aged 3–10 years old	Isolation methods (rubber dam or cotton roll isolation)	Modified Venham scale (hetero-evaluation scale), Oxygen Saturation, Visual Analogue Scale (VAS)	Rubber dam isolation effectively reduces stress in children during dental care.

Kotian et al., 2021 [100]	Questionnaire study	50 children 3–6 years old	Coloring smiley faces, and choosing dental equipment's colors	Modified Child's Dental Anxiety Scale. Color choice	By allowing children to choose their preferred color for the dental environment, the study successfully reduced anxiety levels.

Abdalhai et al., 2024 [102]	Single-blinded RCT	56 children aged 6–10 years old	Aromatherapy with music	Self-report anxiety scale Facial Image Scale (FIS), Face-Legs-Activity-Cry-Consolability (FLACC), heart rate, SpO_2_ saturation, diastolic and systolic blood pressure	It is an effective, low-cost, simple, and safe technique for managing dental anxiety and improving physiological parameters (except SpO_2_ saturation) in children. However, it did not show a significant effect on pain perception.

Rank et al., 2019 [103]	Randomized and blind study	306 children aged 4–6 years old	Positive reinforced technique	Venham Picture Test (VPT)	Awards after dental care reduced anxiety in children.

## Data Availability

Data sharing is not applicable in this study. All data analyzed in this narrative review are from previously published studies; no new datasets were generated.
